# Targeting DNA Repair through Podophyllotoxin and Rutin Formulation in Hematopoietic Radioprotection: An *in Silico, in Vitro, and in Vivo* Study

**DOI:** 10.3389/fphar.2017.00750

**Published:** 2017-10-31

**Authors:** M. H. Yashavarddhan, Sandeep K. Shukla, Pankaj Chaudhary, Nitya N. Srivastava, Jayadev Joshi, Mrutyunjay Suar, Manju L. Gupta

**Affiliations:** ^1^Division of Radioprotective Drug Development Research, Institute of Nuclear Medicine and Allied Sciences, Defence Research and Development Organization, Timarpur, India; ^2^KIIT School of Biotechnology, KIIT University, Bhubaneswar, India; ^3^Centre for Cancer Research and Cell Biology, Queen’s University Belfast, Belfast, United Kingdom; ^4^Department of Pharmacology, University of Illinois at Chicago, Chicago, IL, United States

**Keywords:** DNA damage and repair, cell death, ROS, cell cycle, radioprotection

## Abstract

Drug discovery field has tremendously progressed during last few decades, however, an effective radiation countermeasure agent for the safe administration to the victims of radiation exposure is still unavailable. This multi-model study is aimed at elucidating the mechanistic aspects of a novel podophyllotoxin and rutin combination (henceforth referred as G-003M) in the hematopoietic radioprotection and its involvement in the DNA damage and repair signaling pathways. Using *in silico* study, we identified the binding sites and structural components of G-003M and validated *in vitro*. We further studied various *in vivo* endpoints related to the DNA repair and cell death pathways in mice pre-administered with G-003M, irradiated and subsequently euthanized to collect blood and bone marrow cells. *In silico* study showed the binding of podophyllotoxin to β-tubulin and presence of a functional hydroxyl group in the rutin, suggested their involvement in G2/M arrest and the free radical scavenging respectively. This experimentation was further validated through i*n vitro* studies. *In vivo* mice studies confirmed that G-003M pre-administration attenuated DNA damage and enhanced repair after whole body exposure. We further noticed a decrease in the levels of γH2AX, p53BP1, and ATM kinase and an increase in the levels of DNA pk, Ku 80, Ligase IV, Mre 11, Rad 50 and NBS 1 in the blood and bone marrow cells of the G-003M pre-administered and irradiated mice. We noticed an overall increase in the pro-survival factors in the G-003M pre-treated and irradiated groups establishing the radioprotective efficacy of this formulation. The lead obtained from this study will certainly help in developing this formulation as a safe and effective radioprotector which could be used for humans against any planned or emergency exposure of radiation.

## Introduction

**FIGURE 1:**
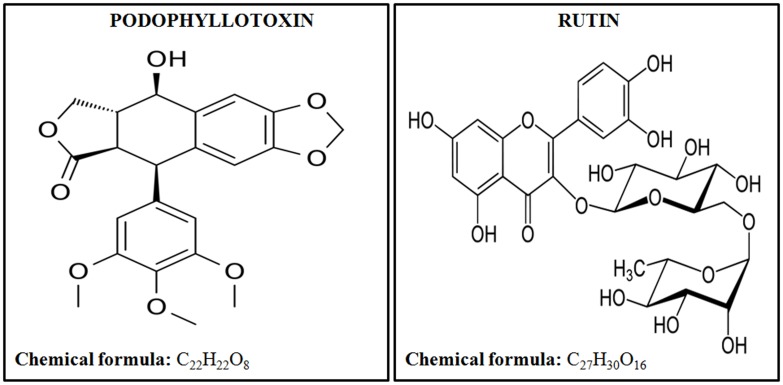
Chemical structures and formulas of podophyllotoxin and rutin.

United States Food and Drug Administration (FDA) has approved only a few radiation countermeasure agents with limited applications suitable for radiation victims. FDA approved safe radioprotector is highly desirable by armed forces and patients undergoing radiotherapy. In this paper we are showing the effectiveness of a novel combination of podophyllotoxin and rutin against low, sub-lethal, and lethal radiation dose. Podophyllotoxin (**Figure 1**) is a lignan, that directly targets DNA topoisomerase-2α (Zhang et al., 1992; Kamal et al., 2005), tubulin alpha-4A chain (Screpanti et al., 2010) and tubulin-β chain (Wolff et al., 1991) confirming its involvement in the mitotic arrest and rejoining of DNA strand breaks (Wolff et al., 1991; Kamal et al., 2005). Podophyllotoxin is further involved in the enzymatic activation of Cytochrome P450-2C19 and P450-3A4 important in NADPH-dependent electron transport pathway (Preissner et al., 2010). Rutin (**Figure 1**) is a flavonoid involved in free radicals scavenging. Rutin targets Aldo-k-keto-reductase-1-member-C3 (Imming et al., 2006) and also activates enzymes Cytochrome-P450-2C8, P450-2C9, P450-2D6 and P450-3A4, which subsequently regulates metabolic processes, cell proliferation and death.

Besides their individual properties, the rationale for combining the podophyllotoxin and rutin is based on earlier observations. Our initial studies using whole and semi-purified fraction of *Podophyllum hexandrum* have demonstrated several properties required for radioprotective action. The various fractions have significant survival efficacy along with protection to hematopoietic, gastrointestinal (GI), bone marrow, immune system and other tissues in lethally irradiated mice (Gupta et al., 2008; Lata et al., 2009; Sankhwar et al., 2011; Dutta et al., 2012). However, the studies carried out in whole and semi purified extracts had major limitations such as presence of large quantity of unidentified molecules which hindered pharmacokinetic and pharmacodynamic studies, essential to understand the mode of action. To overcome these limitations, chemoprofiling led to the identification of various compounds viz, podophyllotoxin, de-metyl podophyllotoxin their glucosides and flavanoids such as quercetin, rutin, kaempferol etc. present in them. Considering the pharmaceutical properties and commercial availability we made a combination podophyllotoxin and rutin which is coded as G-003M. Our group has extensively worked with this combination and published studies showed more than 85% radioprotection considering animal survival as an end-point (Kalita et al., 2016; Yashavarddhan et al., 2016; Singh et al., 2017). Further, this combination has shown radiation dose reduction factor (DRF) of 1.26 and -240 to +10 min therapeutic window (Singh et al., 2017). The combination significantly protected the mice hematopoietic (Yashavarddhan et al., 2016; Singh et al., 2017), gastrointestinal (Kalita et al., 2016) and respiratory systems (Saini et al., 2013) against lethal radiation dose. Our earlier investigations have showed the overall haematopoietic protection by G-003M, however, the mechanistic aspects of the protection to radiation induced haematopoietic damage were largely unknown, which warranted this study.

In the current work we made a detailed investigation of the mechanism of action of this novel combination in the hematopoietic radioprotection by mainly focussing on the DNA damage and Non-homologous end joining repair (Mahaney et al., 2009; Liu et al., 2012). This pathway has been extensively studied for targeting the tumor cells to increase the efficacy of chemotherapy and radiotherapy. After sensing DNA damage, repair responsive proteins like H2AX, 53BP1 and ATM get phosphorylated at the DNA double strand break (DSB) sites during NHEJ pathway that operates in all phases of cell cycle (Tanaka et al., 2006; Mao et al., 2008; Luo et al., 2013; Maréchal and Zou, 2013; Guleria and Chandna, 2016; Pauty et al., 2016). This pathway requires the Ku70/Ku80 hetero-dimer that binds to the DNA ends at DSBs in a sequence-independent manner and plays an important role in DNA repair (Polo and Jackson, 2011). DNA dependent protein kinase (DNA pk) holo-enzyme shows kinase activity and can phosphorylate proteins involved in this pathway (Kanungo, 2013; Davis et al., 2014). Finally, XRCC 4 promotes DNA ligase IV protein which joins the DNA broken ends. Additional processing is performed by the MRN complex (Mre 11, Rad 50, and NBS 1) and Artemis (Chiruvella et al., 2013). The cell death and apoptosis pathway gets activated in the cells with irreparable complex DNA damage. Transcriptional factor, p53, activates various pro-apoptotic proteins involved in cell death process (Belka et al., 2004). The proteins Caspase 3 and BAX also play an important role in the ionizing radiation mediated cell death process (Chong et al., 2000; Bucci et al., 2006). However, anti-apoptotic protein Bcl 2 is another important regulators of cell death pathway (Youle and Andreas, 2008), which has been demonstrated for its inhibitory effect on various pro-apoptotic proteins. We measured both the pro-apoptotic and anti-apoptotic proteins in hematopoietic tissues with this combination. G-003M was administered intramuscularly to the mice before 9 Gy acute total body irradiation. DNA damage accumulation and repair ability of G-003M against lethal radiation exposure was measured through γH2AX, p53BP1, ATM, DNA pk, Ku 80, Ligase IV, Mre 11, Rad 50 and NBS 1 proteins in the blood and bone marrow cells of mice. We further studied the hematopoietic cell regeneration and apoptosis in the both cell types. The observations of the current study affirm the better understanding of the mode of action of G-003M in conferring radiation protection to hematopoietic system.

## Materials and Methods

### Reagents and Cells

Podophyllotoxin (344192), rutin (R5143), sodium chloride (S3014), potassium chloride (P9541), sodium phosphate dibasic (S3264), potassium phosphate monobasic (P9791), potassium bicarbonate (12602), ammonium chloride (A9434), ethylene di amine tetra acetic acid di sodium salt (E6635), paraformaldehyde (158127), poly-L-lysine (P8920), tween 20 (P9416), bovine serum albumin (BSA) (5482), ribonuclease A from bovine pancreas (R6513), propidium iodide solution (P4864), triton X-100 (T8787), DCF-DA (D6883), MTT (T8787), and ethidium bromide solution (E1510) were purchased from Sigma–Aldrich, St. Louis, MO, United States. Dimethyl sulfoxide (DMSO) (16743.0521), methanol (1060091000), ethanol (100983), anti-phospho-histone monoclonal γH2AX Ser 139 (05-636), anti-phospho-ATM (Ser1981) (05-740), Anti-hMre 11 (PC388), Anti-Rad 50 (07-1781) Anti Anti-Cyclin B1 PE (FCMAB102P), goat anti-mouse IgG (H + L) fluorescein-conjugated (AP308F), and goat anti-rabbit IgG (H + L) fluorescein-conjugated (AP307F) were purchased from EMD Millipore, United States and Canada. p53 Binding Protein 1 antibody (PA1-46147), DNA PKcs Antibody (MA5-13404), versene solution (15040066) were purchased from Thermo scientific, United States. Anti-Ku 80 antibody (ab119935), Anti-DNA Ligase IV antibody (ab80514), and Anti-p95 NBS 1 antibody (ab32074) were purchased from Abcam, Cambridge, United Kingdom. DMEM medium (AL007A), trypsine 2.5% solution (TCL032), RPMI-1640 medium (AT028), fetal bovine serum (FBS) (RM10432), dulbecco’s phosphate buffer saline (TS1006), antibiotic and anti micotic solution (A002A), Hi-cDNA synthesis kit (MBT076) and Hi-SYBr master mix with taq polymerase (MBT074) were purchased from Himedia, Mumbai, India., DNAeasy mini kit (69504), and RNeasy mini kit (74104) were purchased from Qiagen, Hilden, Germany. Giemsa stain (G146), and may-Grunwald stain (12608636) were procured from Fisher Scientific. DNA-binding dye “DAPI” (Santa Cruz, CA, United States, Cat No: SC-3598) and primers (IDT Integrated DNA technology, United States), were procured from the specified manufacturers.

1 L 1X PBS (8 g of sodium chloride, 0.2 g of potassium chloride, 1.44 g of sodium phosphate dibasic, 0.25 g of potassium phosphate monobasic to 1 L at pH 7.4), 1 L 1X RBC lysis buffer (1 g of potassium bicarbonate, 8 g of ammonium chloride and 0.03 g of di-sodium EDTA), 10% poly-L-lysine/PBS for slide coating, 3% paraformaldehyde, 50 mM ammonium chloride, 0.1% PBST (0.1% tween 20 in PBS), 1% BSA/0.1% PBST, 70% ethanol in PBS, 1 mg/ml RNase in PBS, wash buffer (2% FBS in PBS), permeabilization buffer (0.25% of triton X-100 in wash buffer) may-grunwald-giemsa stain mixed in 3:1 ratio were prepared in the laboratory.

Human Jurkat cells, and Human HaCaT cells were obtained from National Centre for Cell Science, Pune, India.

### *In Silico* Studies

#### Docking Study

Compounds targeting tubulin have G2/M arresting property. Podophyllotoxin shares structural similarity with colchicine, which targets the β-tubulin and inhibits tubulin assembly for microtubule formation. To understand the binding affinity of podophyllotoxin and colchicine, which is taken as positive control, toward tubulin, a comparative docking study was designed. The study was performed using Autodock Vina software (Trott and Olson, 2010; Chang et al., 2010). A structure of tubulin-α-β-dimer (PubMed: 9428769; PDB ID: 1TUB) (Nogales et al., 1998), co-crystallized with taxotere, a potent inhibitor of tubulin protein, and resolved at 3.70 Angstrom by electron diffraction, was downloaded from the PDB database. The alpha chain was not considered due to the presence of ligand in the beta chain. Protein refinement step was performed prior to the docking studies. Alternative conformation of amino acids, ligands, water molecules and ions were removed. After addition of hydrogen atoms, cleaned beta chain of tubulin protein was converted to PDBQT format, which hold the information of charge for all the atoms. 3D conformations of both the ligands were downloaded from the PubChem data base (Podophyllotoxin ID: 10607, Colchicine ID: 6167). Structures were cleaned, minimized with steepest descent algorithm and finally converted into PDBQT format. Co-crystalized tubulin and native ligand taxotere was docked for optimization of the parameters. After optimization, podophyllotoxin and rutin were docked with tubulin protein using AutodockVina software. Images for protein-ligand interactions were generated with the help of Discovery Studio Visualizer Version 4.

#### Target Identification Using Bioactivity Data Sheet

To assess the off targets for podophyllotoxin and rutin, the bioassay data sheet of both the compounds were downloaded from the PubChem data base (Podophyllotoxin ID: 10607, Colchicine ID: 6167) (Wang et al., 2014). Python script was prepared for automatic analysis of bioactivity data. Inactive targets and repeated entries were further filtered out to achieve the final data sheet which contains the bioactive targets participate in various key biological processes that may contribute in response to radiation injury.

### *In Vitro* Studies

#### Cell Culture

For cytotoxicity Assay, the human keratinocyte cell line HaCaT was cultured in Dulbecco’s Modified Eagle’s Medium with high glucose, L-Glutamine and 10% FBS (fetal bovine serum) containing 100 μl/ml anti-micotic solution. Cells were maintained at 37°C in a humidified atmosphere at 5% CO_2_ condition. The cell-cycle analysis was done in human Jurkat T-cells cultured in RPMI-1640-medium supplemented with 10% fetal bovine serum and 100 μl/ml anti-micotic solutions at 37°C in a humidified atmosphere with 5% CO_2_.

#### Cyto-Toxicity Assay (MTT)

The HaCaT cells (10^4^/well) were seeded in 96 well plate were incubated overnight subsequently, cells were treated for 24 h with different concentrations (0.025, 0.05, 0.1, 0.15, 0.2, 0.25, 0.3, and 0.4 μg/ml of cell culture medium) of podophyllotoxin alone, rutin alone, and in combination of both (G-003M). After 24 h of incubation of these compounds, cells were incubated with 20 μl of MTT (5 mg/ml in Dulbecco’s Phosphate Buffer Saline) for 4 h on 37°C. Absorbance was recorded at 570–630 nm by dissolving resultant formazon with 100% DMSO.

#### Cell-Cycle Analysis and Cyclin B1 Expression Study

Jurkat cells seeded in T-25 flask (1 × 10^6^ cells/ culture) with 5 ml of complete medium were incubated at 37°C in a humidified atmosphere with 5% CO_2_ condition in incubator for overnight. Cells were treated with 2.0 μg/ml (Based on the MTT assay results determined as the effective and safe concentration) of podophyllotoxin for 1hr and medium was replaced with the fresh media. The cultures were fixed at 4, 6, 8, and 24 h time points.

#### Cell-Cycle Analysis

For cell-cycle analysis study Jurkat cells from different treated groups (unstained, untreated, podophyllotoxin alone) after exposing to different time point (4, 6, 8, and 24 h time points) were analyzed using flow-cytometer. Briefly, the cells were washed thrice in ice-cold PBS, fixed in chilled 70% ethanol and kept overnight at -20°C. Approximately one million cells from each sample were washed with PBS (phosphate buffer saline), suspended in 1 ml PBS containing 200 μg RNase for 45 min at 37°C. Cells were then incubated with propidium iodide (50 μg/ml) on ice for 30 min in the dark. From each sample 10000 cells were acquired on a BD LSR II cytometer (BD Biosciences, San Jose, CA, United States) equipped with 485 nm excitation source and 620 nm emission filters at a flow rate of 200 events/s.

#### Measurement of Cyclin B1

Jurkat cells from different treated groups (unstained, untreated, podophyllotoxin alone) at various time points (4, 6, 8, and 24 h time points) were analyzed flow-cytometrically. Briefly, following three washes in ice-cold wash buffer (2% FBS in PBS) cells were fixed with chilled 70% alcohol and kept at -20°C overnight. Ethanol fixed cells were permeabilized by permeabilization buffer. After the permeabilization process, cells were stained with anti cyclin B1 PE conjugated antibody (1:200 dilutions). Cells were washed three times with wash buffer. 10000 cells/sample were acquired using BD LSR II (BD Biosciences, San Jose, CA, United States) equipped with 485 nm excitation source and 620 nm emission filters at a flow rate of 200 events/s.

### *In Vivo* Studies

#### Animals

All animal care and experimental protocols were approved by the committee on the Ethics of Animal Experiments of the Institute of Nuclear Medicine and Allied Sciences (INMAS), Defence Research and Development Organizations (DRDO), Delhi, India (Institute Animals Ethics Committee number: INM/IAEC/2016/21 valid until 23/02/2017). Animal studies are reported in compliance with the ARRIVE guidelines (Kilkenny et al., 2012). Eight week old Strain ‘A’ male mice (26–30 g weight) were taken from Experimental Animal Facility Institute of Nuclear Medicine and Allied Sciences (INMAS). Mice were housed for a week in a stainless cage under conventional conditions for acclimatization. They were kept at a constant temperature of 25 ± 3° C and relative humidity of 30–70% under a 12 h light/dark cycle. Food and water were available *ad libitum*.

#### Treatment Groups

The animals were assigned to one of the following treatment groups: Untreated, G-003M alone, Radiation alone and G-003M pre-treated and irradiated.

#### Podophyllotoxin and Rutin Combination Preparation and Administration

Podophyllotoxin and rutin were mixed at a 1:1 ratio with 5% DMSO as the solvent. The formulation was diluted in double distilled water and injected intramuscularly into mice 1 h before gamma irradiation (Yashavarddhan et al., 2016). 2.5 mg/kg body weight concentration of the combination as a prophylactic single dose was administered intramuscularly using sterile 26 G needle.

#### Radiation Treatment

Mice were exposed to lethal dose 9 Gy of total body gamma irradiation in cobalt ^60^ (Co^60^) tele therapy unit at fixed dose rate of 1.0 Gy/min under the supervision of a Radiation Safety Officer. After irradiation animals were kept in cages and euthanized at various time points according to the experimental design.

#### Blood and Bone Marrow Cells Collection

Different groups of mice were euthanized at various time points using cervical dislocation and the blood and bone marrow cells were collected for further study. One milliliter blood was collected from the heart by cardiac puncture in EDTA tubes to avoid clotting. The collected blood from each experimental animal was taken for hematology and isolation of blood leucocytes. The leucocytes were isolated from the blood by removing RBCs with the help of RBCs lysis buffer. Marrow cells were flush out from the femur bone in 1ml of 1X PBS and the RBCs were removed using RBCs lysis buffer.

#### Blood Hematology

A 200 μl sample of blood was used to assess hematological parameters from different treatment groups at different time intervals like 1, 3, 7, 15, 20, and 30 days post irradiation. The blood was analyzed using an automated five parts hematology analyzer (ADVIA 2120, Siemens).

#### Bone Marrow Cell Counting

RBC lysed bone marrow cells from different treatment groups were counted using Neubauer’s chamber after appropriate dilution under 20X objective fields of an upright bright field microscope (Olympus BX 53) at 6 h, 1, 7, 15, and 30 day time intervals.

#### Preparations of Bone Marrow Smear

The collected bone marrow cells at 6 h, 1, 7, 15, and 30 day time points from different treatment groups were centrifuged at 2000 rpm 8 min and re-suspended in 50 μl of FBS. A small drop of cells was put on a clean microscopic slide and smeared over an area of 2–3 cm by pulling another glass slide held at a 45° angle. Cells were fixed with methanol and stained with may-grunwald giemsa stain. Random 1000 cells were counted using upright bright field microscope from the thin portion of each slide.

#### Immunocytochemistry Study

The study was designed to evaluate the modulatory effect of G-003M on radiation induced DNA damage/repair proteins at various time points. For γH2AX, p53BP1, the DNA DSBs biomarkers, 1, 2, and 24 h time points were chosen and for other proteins like, ATM, DNA pk, Ku 80, Ligase IV, Mre 11, Rad 50 and NBS 1, the time points chosen were 30, 60, 180, and 360 min. The RBC lysed blood and bone marrow cells were fixed using 4% formaldehyde/PBS for 30 min on ice and then spotted at a concentration of 1 × 10^5^ cells/ml onto Poly-L-Lysine coated glass. Un-lysed RBCs were removed using 50 mM of ammonium chloride. Thereafter, cells were incubated in 1% BSA in 0.1% PBS-Tween for 1 h to permeabilized the cells and block non-specific protein-protein interactions. After blocking, cells were incubated overnight with γH2AX (dilution 1:500), p53BP1 (dilution 1:500), ATM (dilution 1:500), DNA pk (dilution 1:500), Ku 80 (dilution 1:500), Ligase IV (dilution 1:500), Mre 11 (dilution 1:500), NBS 1 (dilution 1:500) and Rad 50 (dilution 1:500) for 120 min at room temperature. Cells were washed and incubated over night with appropriate FITC (Fluorescein isothiocyanate)-conjugated secondary antibody with 1:1000 dilutions in PBST at 4°C. After washing, the slides were incubated with 200 ng/ml DAPI in PBS for 30 min at room temperature. The slides were then washed, dried and mounted. Foci were scored under an upright epi-fluorescence microscope (Olympus BX 63) using a 100X objective. Five hundred cells were scored for each experimental group.

#### Flow Cytometric Measurement of γH2AX, and P53BP1 in Mice Blood and Bone Marrow Cells

Flow cytometric measurements were used to validate the γH2AX, and p53BP1 foci data obtained from Immunocytochemistry study. The animals were exposed to a dose of 9 Gy using Co^60^ teletherapy unit and γH2AX, and p53BP1 mean fluorescent intensity was measured at 1 h time point in blood and bone marrow cells. RBC lysed blood and bone marrow cells were washed twice with PBS (centrifugation at 1200 rpm for 10 min) and fixed in 100 μl of 3% paraformaldehyde for 30 min on ice. The fixed cells were washed twice with 1 ml of PBS followed by washing with 1 ml of 50 mM ammonium chloride. After washing twice with PBS, the cells were permeabilized in 200 μl of 0.1% Triton X-100 in PBS for 20 min at 4°C. The blocking step was performed in 10% BSA in PBS for 100 min at room temperature, followed by an overnight incubation with anti-phospho-histone γH2AX Ser-139 monoclonal antibody (dilution 1:50) and p53 Binding Protein 1 antibody (dilution 1:50) as per the experimental design. The cells were then washed with PBS and incubated with polyclonal goat anti-mouse FITC-conjugated secondary antibodies (1:400) at 4°C. The fluorescence intensity of γH2AX, and p53BP1in 10,000 cells/sample were analyzed using a BD flow cytometer (Srivastava et al., 2014).

#### DNA Fragmentation Assay in Blood and Bone Marrow Cells

The experiment was designed to study the radiation induced apoptosis in the form of DNA ladder in blood and bone maarow cells of mice in different treatment group at 6 h time point. The RBC lysed cells were fixed with 70% ethanol and stored overnight at -20°C. Total DNA was isolated from the ethanol fixed 1 × 10^6^ cells using “DNeasy Blood & Tissue Kits” acquired from QIAGEN (according to the manufacturer’s protocol). DNA quantification was done using UV visible spectrophotometer (DU Series 600 spectrophotometer) from Beckman Coulter. Five microgram of extracted DNA was mixed with 3 μl of DNA Gel Loading Dye (6X) from Thermo Fisher Scientific and electrophoresed on a 1.2% agarose gel at 100 v for 30 min on Biorad Horizontal electrophoresis unit. The gel was visualized by UV light after standard ethidium bromide staining. The gels were analyzed by Gel-analyzer software.

#### RNA Isolation, Quantification and Quantitative RT-PCR Analysis

To corroborate the immunofluorescence and cell death experiments, Q-PCR study was designed to evaluate the modulatory effect of G-003M on radiation induced DNA damage/repair and cell death genes in different treatment groups. For DNA damage/repair genes ATM, ku 80 Ligase IV and Mre 11 along with the housekeeping gene β-actin were measured at 1 h of post irradiation time for different experimental groups. P53, caspase 3, BAX, and BCL 2, the cell death genes were evaluated after 3, 6, and 24 h of radiation. RNA was isolated from 70% ethanol fixed samples for DNA damage/repair and cell death related genes of mouse blood and bone marrow cells using Qiagen blood and tissue Kit as per the manufacturer’s protocol. RNA concentration and purity were estimated using Nano Drop 2000c (Thermo fisher).

For gene expression study, primers for β-actin, ATM, Ku 80, Ligase IV, Mre 11, P53, Caspase 3, BAX and BCL 2 were designed using NCBI primer designing software and purchased from IDT Integrated DNA technology, United States company. The sequence and annealing temperature of each primer was mentioned in **Table 1**. The Hi-cDNA synthesis kit was used for cDNA synthesis from 50 ng of total RNA according to the manufacturer’s protocol. The Q-PCR reactions were prepared using Hi-SYBr Master Mix by following the instruction provided by the manufacturer. The samples were analyzed using INSTA Q-96 PCR machine from Himedia, India. The optimized PCR cycling conditions were as follows: Initial denaturation 94°C for 5 min, denaturation 94°C for 30 s and annealing as specified in **Table 1** for 30 s.

**Table 1:** Primers detail.

Gene	Accession no	Primer sequence	Annealing temperature
β-actin	NM_007393.5	FP- TGGAATCCTGTGGCATCCATGAAAC	56°C
		RP- TAAAACGCAGCTCAGTAACAGTCCG	
ATM	NM_007499.2	FP- ATATGCCAGTCTTTTCAGGGTG	56°C
		RP- GCGCTCTCTGTCTGTGACTG	
Ku 80/ XRCC 5	NM_009533.2	FP- CATGGCGTGGTCCGGTAATA	56°C
		RP- GCTCTCCGAAAATACCTGTCG	
Ligase IV	NM_176953.3	FP- GCTCGCTGGGGACTGATTTC	56°C
		RP- CGGTTTGAACTAAGCAGCCC	
Mre 11	NM_018736.3	FP- TCTGGGAGCGGTTTTCTTGT	56°C
		RP- CATCGTCAAGTGGATCTGTGG	
p53	NM_011640.3	FP- GTGCTCACCCTGGCTAAAGT	57°C
		RP- GCAGTCATCCAGTCTTCGGA	
Caspase 3	NM_001284409.1	FP- GAGCTTGGAACGGTACGCTAA	57°C
		RP- CCCAGAGTCCACTGACTTGC	
BAX	NM_007527.3	FP- CTCAAGGCCCTGTGCACTAA	57°C
		RP- GAGGCCTTCCCAGCCAC	
BCL 2	NM_009741.5	FP- AGGCTGGGATGCCTTTGTGG	57°C
		RP- ACTTGTGGCCCAGGTATGC	


*FP, forward primer; RP, reverse primer.*

#### Determination of Reactive Oxygen Species (ROS)

Reactive oxygen species levels in the mouse blood and bone marrow cells at 1 h time point were determined by DCF-DA fluorescent assay with minor modifications. Animals were divided into following groups- untreated, rutin alone, radiation alone and Rutin + radiation. After radiation exposure, blood and femur bones were excised from the animals. RBC were lysed in the isolated cells and cells were counted using a haemocytometer. One million cells/ml was incubated with 10 μmol/L DCF-DA at 37°C for 30 min. After incubation, cells were analyzed by BD flow-cytometry using 10,000 events/sample.

### Data Analysis

The data obtained are represented as mean ± SD. The difference between the experimental groups was evaluated by one-way analysis of variance, with Newman–Keuls multiple comparison test (V, 5.01; Graph Pad Prism, San Diego, CA, United States). Comparisons were made among the untreated, radiation, G-003M alone and radiation + G-003M groups for immunocytochemistry, DNA fragmentation, blood hematology, bone marrow smear and counting studies. In ROS study the untreated, radiation, Rutin alone and Rutin + radiation groups were compared. Similarly in MTT assay study untreated DMSO alone, podophyllotoxin alone, rutin alone and G-003M alone was compared. For cell-cycle study comparisons were made among the untreated, podophyllotoxin alone. In case of DNA pk, Ku 80, Ligase IV, Mre 11, NBS 1 and Rad 50 heat map analysis was done by using R-python software. All experiments consisted of 6 animals and each experiment run in duplicate. A value of *p* < 0.05 is considered statistically significant. ^∗^*p* < 0.05, ^∗∗^*p* < 0.01, ^∗∗∗^*p* < 0.001, ^ns^not significant (*p* > 0.05).

## Results

### Mode of Action of G-003M

We have investigated the mode of action of G-003M by analyzing the property of individual compounds, i.e., podophyllotoxin and rutin with *in silico, in vitro* and *in vivo* methods.

#### Properties of Podophyllotoxin and Rutin

**FIGURE 2:**
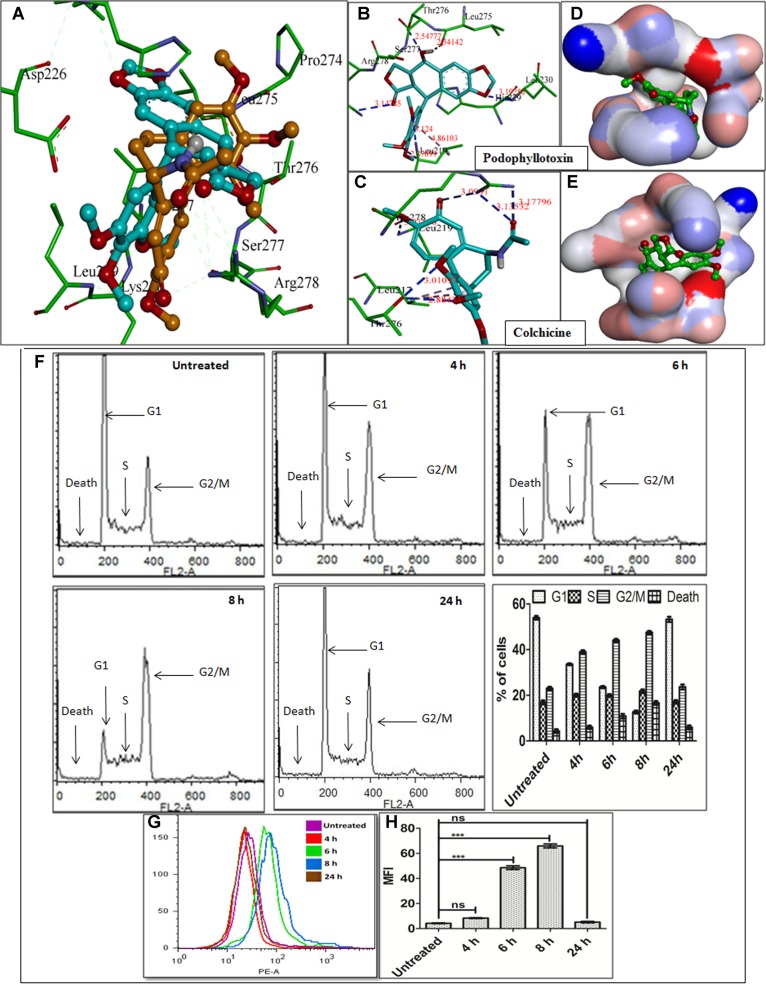
Comparative docking study of podophyllotoxin and colchicine: **(A)** Docked and superimposed conformations of both, podophyllotoxin (cyan) and colchicine (orange) were represented as Ball and stick model along with the β-tubulin protein residues (green) as stick model. **(B,C)** Interaction of both podophyllotoxin and colchicin with Binding site residues were represented. **(D,E)** Charge distribution within the binding pocket was depicted. G2/M arrest by podophyllotoxin in human jurkat cells: Cells were treated with podophyllotoxin for 1 h and cell cycle analysis was performed by propidium iodide method. **(F)** The histogram represents the different phases of cell-cycle. The bar graph represents percentage of cells in different phages of cell-cycle at 4, 6, 8, and 24 h time points. The bar chart shows the mean ± SD of 6 replicates**. (G)** The histogram and graph indicate the Cyclin B1 expression study in jurkat cells at different time points. **(H)** The bars in graph represents mean fluorescent intensity of Cyclin B1 at different time points. *n* = 6 per group.

An *in silico* study was designed using PubChem bioactivity data base to investigate the off-targets of podophyllotoxin. Here, we identified, 61 unique and important off-targets of podophyllotoxin involved in radiation response manifestation like inflammation, DNA repair, cellular growth, differentiation, neuronal function etc. as shown in Supplementary Table 1. Podophyllotoxin is a known G2/M cell-cycle arresting compound possibly due to its binding with β-tubulin protein which was observed as an important off-target in our bioactivity assay. Colchicine, a cytotoxic agent, also shares the similar binding site of β-tubulin with podophyllotoxin rasing a question whether this binding is cytotoxic or cytoprotective in nature. To answer this, a comparative docking study was performed for podophyllotoxin and colchicines (**Figure 2A**) using the software Autodock Vina. Interaction of taxotere and tubulin revealed that binding site residues Gly-370 and Thry-276 were involved in H-boding with the ligand, on the other hand, residue His-229, Phe-272, Pro-360, and Val-238 were involved in weak interactions. Docked conformation of colchicines and podophyllotoxin have adopted similar orientation as taxotere, where residues, Leu-219, Leu-275, Arg-278 and Thr-276, were involved in H-Bonds, while residue Leu-217 and Leu-219 involved in hydrophobic interactions (**Figures 2B,D**). Colchicine and podophyllotoxin form six and five H-bonds respectively with tubulin protein. Podophyllotoxin binds little bit deeper into the pocket due to its extra aromatic ring. Bigger size of Podophyllotoxin is responsible for its higher binding energy (-8.0 Kcal/mol) as compared to colchicine (-7.6 Kcal/mol) (**Figures 2C,E** and **Table 2**). An extra H-bond and additional hydrophobic interactions provide a higher stable conformation to colchicine in compare to Podophyllotoxin with tubulin protein.

**Table 2:** Non-covalent interaction map.

Interaction type	Distance (Å)	Donor atom/Group		Acceptor atom/Group	
**Podophyllotoxin-tubulin Interaction**					
Hydrogen bond	2.34	N:LIG:H	H-Donor	B:LEU275:O	H-Acceptor
Hydrogen bond	3.12	B:LEU219:N	H-Donor	N:LIG:O	H-Acceptor
Hydrogen bond	2.54	B:THR276:OG1	H-Donor	N:LIG:O	H-Acceptor
Hydrogen bond	3.14	B:ARG278:NH2	H-Donor	N:LIG:O	H-Acceptor
Hydrogen bond	2.87	B:LEU219:CA	H-Donor	N:LIG:O	H-Acceptor
Hydrophobic	4.86	N:LIG	Pi-Orbitals	B:LEU219	Alkyl
**Colchicine-tubulin interaction**					
Hydrogen bond	3.01158	B:LEU219:N	H-Donor	N:LIG:O	H-Acceptor
Hydrogen bond	2.88433	B:THR276:OG1	H-Donor	N:LIG:O	H-Acceptor
Hydrogen bond	3.01097	B:THR276:OG1	H-Donor	N:LIG:O	H-Acceptor
Hydrogen bond	3.13532	B:ARG278:NH1	H-Donor	N:LIG:O	H-Acceptor
Hydrogen bond	3.0991	B:ARG278:NH1	H-Donor	N:LIG:O	H-Acceptor
Hydrogen bond	3.17796	B:ARG278:NH2	H-Donor	N:LIG:O	H-Acceptor
Hydrophobic	4.36313	N:LIG	Alkyl	B:LEU217	Alkyl
Hydrophobic	5.24814	N:LIG	Pi-Orbitals	B:LEU217	Alkyl


To establish the finding of cell-cycle arrest property of the compound by binding with tubulin protein an *in vitro* study was carried out in jurkat cells at different time intervals, i.e., 4, 6, 8, and 24 h. A safe concentration of podophyllotoxin, i.e., 0.2 μg/ml was determined through MTT assay as shown in Supplementary Figure 1, was used. Cell-cycle analysis showed that cells in the G1, G2 and S phases were 55, 23, and 17% respectively in untreated samples. Four hours after podophyllotoxin treatment percentage of cells in G2 phase doubled as compared to the untreated samples and kept on increasing by 6 and 8 h and highest percentage in G2 phase was seen at 8 h. After 24 h the cells were completely released from G2/M block indicating temporary arrest behavior (**Figure 2F**). We further measured the cell-cycle proteins such as Cyclin B1 involved in G2/M arrest in jurkat cells treated with 0.2 μg/ml at various time points (4, 6, 8, and 24 h) as shown in **Figures 2G,H**. We observed a time dependent increase in the expression of cyclin B1 as compared to untreated cells, with highest expression at 8 h that reduced to background levels at 24 h (**Figure 2H**). We have not studied the ROS scavenging ability of the podophyllotoxin in the current study as this compound has not shown significant reduction in radiation induced ROS (Dutta et al., 2015).

**FIGURE 3:**
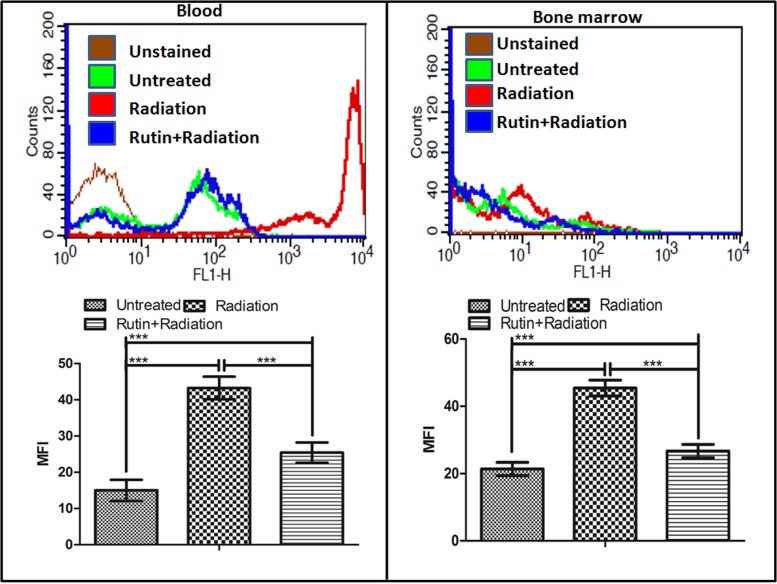
ROS scavenging ability of rutin in the blood and bone marrow cells: The graph and the histogram shows the radiation induced ROS scavenging property of the compound rutin at 1 h time point in blood and bone marrow cells of mice. *n* = 6 per group.

Similar to podophyllotoxin the bioactivity assay of rutin also showed 24 unique off-targets involved in different cellular process correlated in radiation response manifestation like inflammation, DNA repair, cellular growth, differentiation, neuronal function (Supplementary Table 2). Presence of a hydroxyl group in the rutin structure indicate its involvement in free radical scavenging specifically the ROS which we validated in blood and bone marrow cells of mice after radiation as shown in **Figure 3**. Radiation induced significant ROS generation in blood and bone marrow cells compared to untreated control. However, rutin treated and irradiated groups showed reduced levels of ROS generation in both cell types. An overlay of the histograms of different groups clearly indicates that rutin hydrate pre-treatment reduced ROS levels at 1 h time point. We have also treated cells with rutin alone for cell cycle experiment and could not found any cell cycle arresting ability.

### G-003M Reduces Radiation Induced DNA DSBs Formation

**FIGURE 4:**
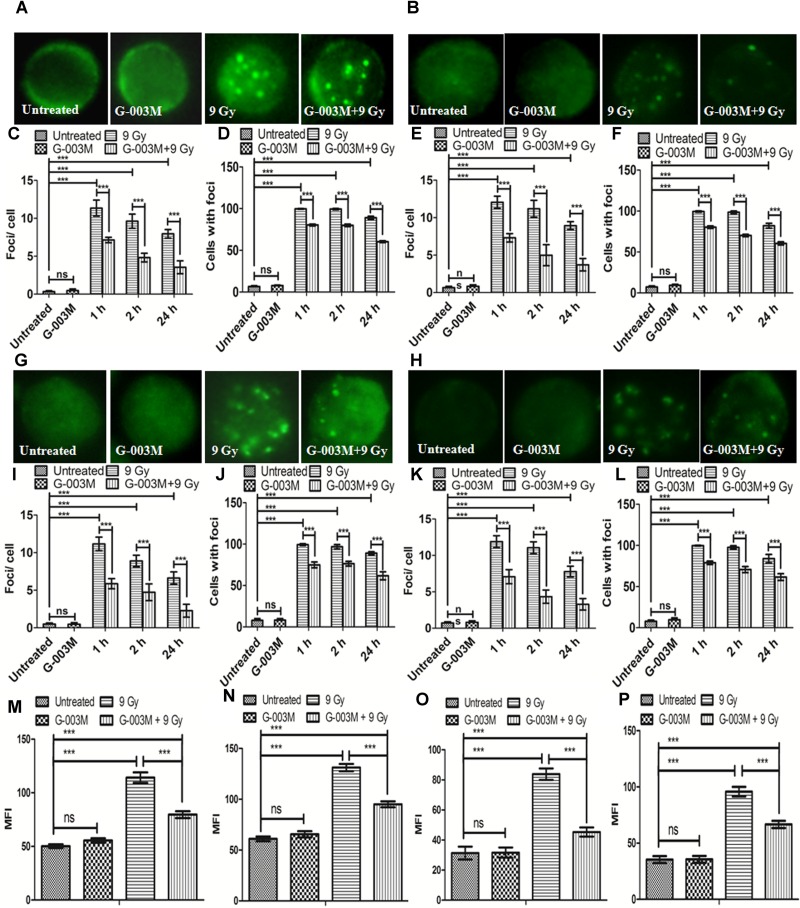
Analysis of γH2AX and p53BP1 foci in blood and bone marrow populations at various times after exposure of mice to ionizing radiation: **(A,B)** Represent assessment of γH2AX foci in the blood and bone marrow respectively. **(C)** Average γH2AX foci per cell **(D)** γH2AX foci distribution in blood cells. **(E)** Average γH2AX foci/ cell **(F)** γH2AX foci distribution in bone marrow cells. **(G,H)** p53BP1 foci induction in the blood and bone marrow samples respectively. **(I)** Average p53BP1 foci per cell in blood **(J)** p53BP1 foci distribution in blood cells. **(K)** Graphs showing the mean p53BP1 foci per cell in bone marrow cells and **(L)** foci distribution in bone marrow cells. **(M)** Graph representing the mean fluorescence intensity of γH2AX in blood cells. **(N)** Graph showing the mean fluorescence intensity of γH2AX in bone marrow cells. **(O)** Graph representing the mean fluorescence intensity of p53BP1 in blood cells. **(P)** Graph showing the mean fluorescence intensity of p53BP1 in bone marrow cells. *n* = 6 per group.

γH2AX and p53BP1 foci formation in the blood and bone marrow cells of mice after whole body irradiation was used to measure DNA DSB formation at various time points, i.e., 1, 2, and 24 h. We noticed significant changes in the distribution of γH2AX and p53BP1 foci after irradiation in both blood and bone marrow cells as compared with the untreated samples (**Figure 4**). γH2AX, and p53BP1 foci number was highest at 1 h post-irradiation, however, in G-003M pre-treated group a significant reduction in foci number was noticed compared to the groups that received radiation alone irrespective of the cell lineage. An overall significant increase in the foci positive cells was seen at all time intervals in the irradiated group compared to untreated, with almost 100% of the cells were found γH2AX and p53BP1 foci positive at 1 h post-irradiation in both cell types. However, G-003M pre-treated and irradiated group displayed significantly decreased percentage of cells with foci compared to the radiation alone group while the cells treated with G-003M alone showed similar foci distribution to the untreated samples (**Figure 4**). We further validated our findings by measuring γH2AX and p53BP1 using flow-cytometry technique in both blood and bone marrow cells of mice at 1 h time point which is the time point where these two proteins have maximum level of expression in our immunocytochemistry study (**Figures 4M–P**). In case of radiation alone (9 Gy) both the proteins have shown significant increase in mean fluorescence intensity in both blood and bone marrow cells when compared with the untreated samples. However, significant reduction in mean fluorescence intensity was observed in the case of G-003M pre-treated and irradiated group in both blood and bone marrow cells as compared to respective radiation alone (**Figures 4M–P**). In both the cell lineages G-003M alone did not show significant changes in MFI when compared with the untreated samples (**Figures 4M–P**). The concentration of G-003M used for the current investigation is safe and did not induce any DSBs as analyzed by measuring γH2AX and p53BP1. This fact has already been published by our group (Srivastava et al., 2014; Yashavarddhan et al., 2016).

**FIGURE 5:**
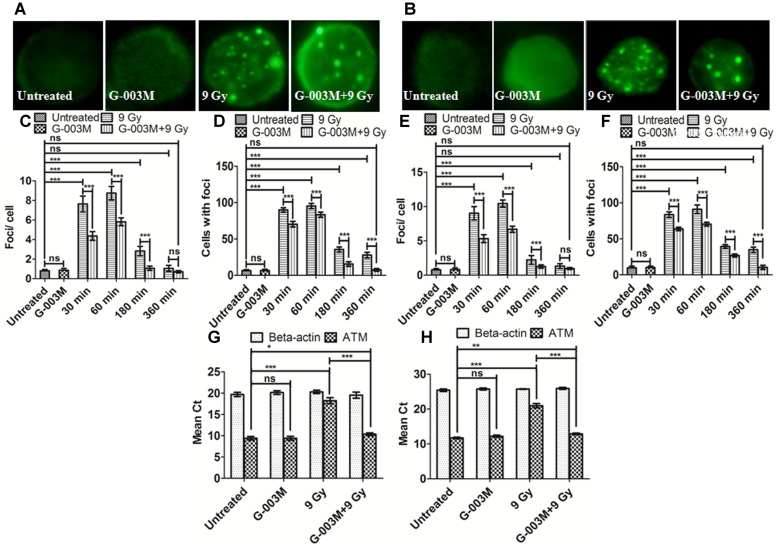
Measurement of ATM kinase in mice blood and bone marrow populations taken at various times after irradiation: **(A,B)** Representative images of ATM kinase foci in blood and bone marrow respectively. **(C)** Quantification of ATM kinase foci per cell and **(D)** ATM kinase foci distribution in blood cells. **(E)** The graph shows mean ATM kinase foci per cell and **(F)** the ATM kinase foci distribution in bone marrow cells. **(G)** The graph represents a quantitative real time PCR bases estimation of β-actin and ATM gene respectively in blood cells processed 1 h after irradiation. **(H)** The graph represents a quantitative real time PCR bases estimation of β-actin and ATM gene respectively in bone marrow cells processed 1 h after irradiation. *n* = 6 per group.

As ATM is an important upstream signaling protein that phosphorylates γH2AX and p53BP1 in the NHEJ pathway we investigated the expression level of ATM ser-1981 in the blood and bone marrow cells of mice at 0.5, 1, 3, and 6 h post-irradiation and noticed that ATM foci numbers reached maximum at 1 h post exposure which could not be seen at the later time points as shown in **Figure 5**. However, an overall significant increase in the foci per cell was observed at all the time points with the only exception at 6 h post-irradiation in comparison with the untreated samples. G-003M alone did not induce any significant changes either in the average foci number or in the foci distribution as compared to the untreated groups (**Figure 5**). However, in the G-003M pre-treated-irradiated group a significant reduction in the average foci number at 0.5, 1, and 3 h was noticed in comparison to the radiation alone group. Irradiated groups have indicated 90% of cells with foci at 1 h. However, time dependent decrease in foci distribution was observed at later time points in both the groups. G-003M treated and irradiated group showed a significantly reduced foci distribution at all the time points in comparison with respective radiation alone (**Figure 5**). We further validated our findings at genomic level by measuring ATM gene expression in both the blood and bone marrow cells of mice using Q-PCR method taking β-actin as houskeeping gene at 1 h time interval (**Figures 5G,H**). The mean Ct values significantly increased in the radiation alone group compared to untreated groups. The Ct values were comparable between untreated and G-003M alone groups. The pre-treatment of G-003M significantly down regulated ATM expression (**Figures 5G,H**).

### G-003M Enhances the DNA DSBs Repair in All the Phases of Cell-Cycle

**FIGURE 6:**
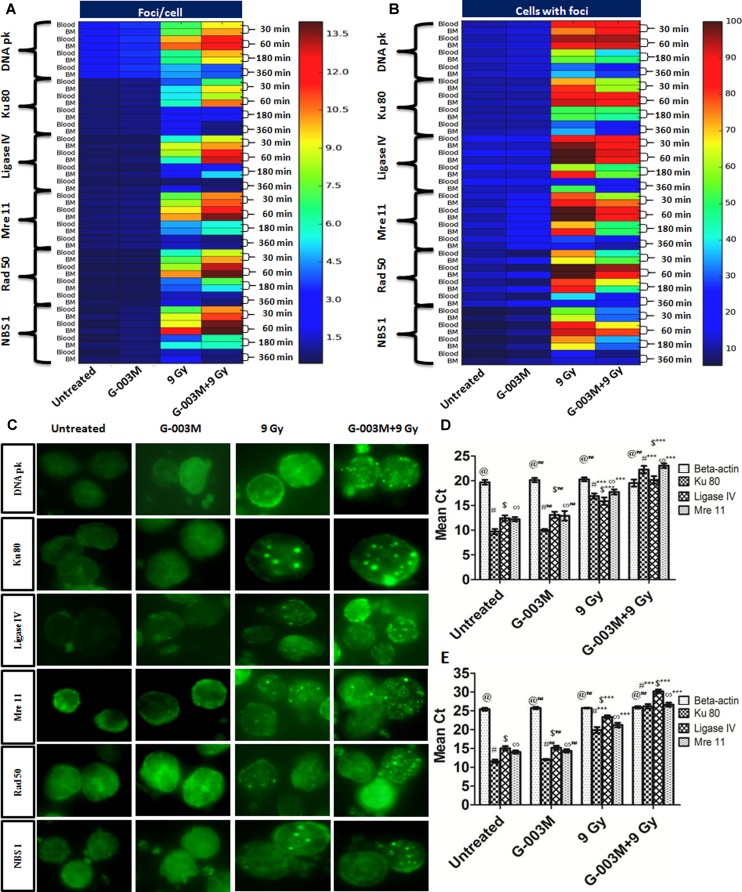
Expression of NHEJ pathway proteins in mice blood and bone marrow populations observed at various times after exposure in the different treatment groups: **(A)** The heat-map represents the foci number of DNA pk, Ku 80, Ligase IV, MRE 11, Rad 50 and NBS 1at different treatment group at different time interval, i.e., 30, 60, 180, and 360 min and **(B)** represents the foci distribution of each protein in the blood and bone marrow cells. **(C)** Representative images of radiation induced foci formation in different treatment group. **(D)** Graphs represent quantitative real time PCR analysis of Ku 80, Ligase IV and Mre 11 along with the housekeeping gene (β-actin) at 1 h time points in blood. **(E)** Graphs represent quantitative real time PCR analysis of Ku 80, Ligase IV and Mre 11 along with the housekeeping gene β-actin at 1 h time points in bone marrow cells of mice. *n* = 6 per group.

G-003M induced cell-cycle changes warranted us to study its effects on the cell-cycle responsive NHEJ pathway proteins. For this experiment we measured DNA pk, Ku 80, Ligase IV, Mre 11, Rad 50 and NBS 1 foci at 0.5, 1, 3, and 6 h post-irradiation. G-003M alone did not induce any foci formation of any of the proteins when compared with the untreated samples (**Figure 6**). In radiation alone group significant increase in foci number and foci positive cells was seen at 0.5, 1, and 3 h point compared to untreated groups. No significant changes between radiation, 6 h and untreated groups with aspect to foci number were noticed however, G-003M treated and irradiated groups showed significant increase in foci number at 0.5, 1, and 3 h as shown in **Figures 6A–C**. The number of foci was higher in G-003M pretreated and irradiated group at 1 h with comparison to radiation alone (**Figure 6**). The foci numbers declined after 1 h post-irradiation in both treatment groups. Percentage of cells with foci had more than 90% of cells with foci at 1 h in both radiation alone and G-003M pre-treated groups. Significant attenuation in percentage of damaged cells was observed in G-003M treated and irradiated group compared to radiation alone at 3 and 6 h (**Figure 6**). Genomic validation of the observed changes in the foci number of these proteins was carried out using Q-PCR by measuring the genomic profile of Ku 80, Ligase IV, and Mre 11 gene at 1h time point, where β-actin gene was used as a housekeeping gene (**Figures 6D,E**). In the radiation-alone group, the mean Ct values significantly increased as compared to untreated samples. The pre-treatment of G-003M further up-regulated the expression of afore-mentioned genes than radiation alone group, however, the Ct values were quite comparable between untreated and G-003M alone groups.

### G-003M Targets the Cell Death Pathway and Reduces the Apoptosis

**FIGURE 7:**
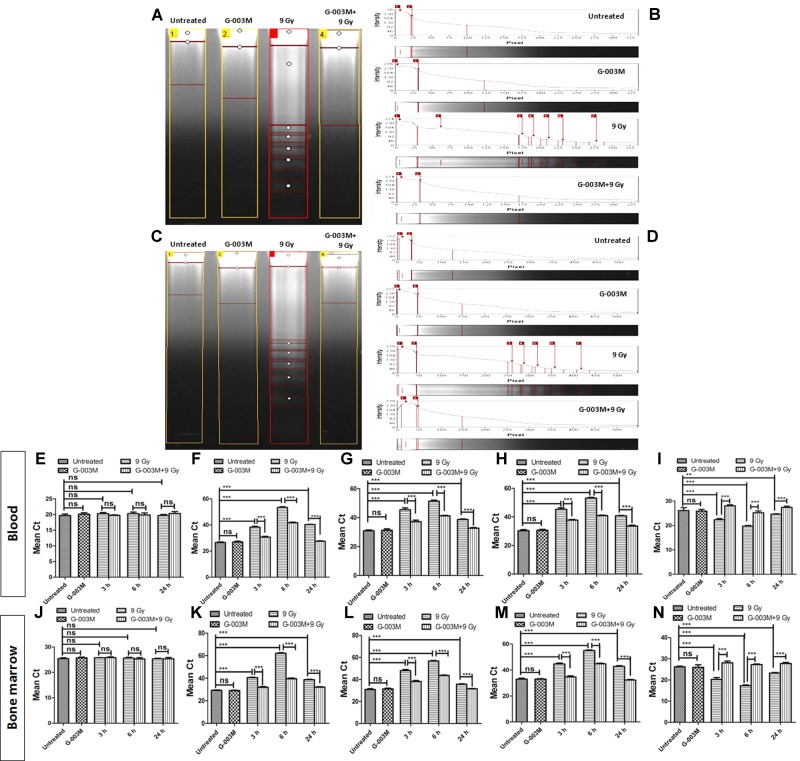
Apoptosis measurement after 9 Gy exposure and its modulation by the combination G-003M: **(A)** DNA fragmentation of different treatment groups at 6 h time points in blood cells. **(B)** The histogram represents the individual lanes with the band intensity of fragmented genomic DNA from blood cells. **(C)** DNA fragmentation of different treatment groups at 6 h time points in bone marrow cells **(D)** The histogram represents the individual lanes with the band intensity of fragmented genomic DNA from bone-marrow cells. **(E–I)** represent quantitative real time PCR analysis of β-actin, p53, Caspase 3, BAX and BCL 2 along at 3, 6, and 24 h time points in blood cells respectively. **(J–N)** represent quantitative real time PCR analysis of p53, Caspase 3, BAX and BCL 2 at 3, 6, and 24 h time points bone marrow cells. *n* = 6 per group.

Radiation-induced apoptosis inhibition by G-003M was studied using DNA fragmentation assay in the blood and bone marrow cells of mice at 6 h post-irradiation (**Figures 7A–D**). The fragmented DNA ladder in radiation alone group can be clearly seen in **Figure 7** (lane-3). No DNA fragmentation was observed in case of untreated G-003M alone, and G-003M pre-treated and irradiated groups, indicating significant reduction in apoptotic cell population in G-003M treated and irradiated groups. Further we have studied the effects of G-003M upon the genomic expression of apoptotic pathway proteins P53, Caspase 3, BAX and BCL 2 at 3, 6, and 24 h post-irradiation in the blood and bone marrow cells by Q-PCR using β-actin gene as a housekeeping gene (**Figures 7E–N**). In radiation alone group the mean Ct values were significantly higher for all the pro-apoptotic genes: P53, caspase 3 and BAX with lower levels of anti-apoptotic gene: Bcl-2 at all the time points when compared with untreated samples (**Figures 7E–N**). The Ct values were comparable between untreated and G-003M alone groups. The pre-treatment of G-003M significantly down regulated the expression of P53, caspase 3 and BAX and up-regulated the expression of BCL 2 gene (**Figures 7E–N**).

### G-003M Maintains Hematopoietic System Cellularity

**FIGURE 8:**
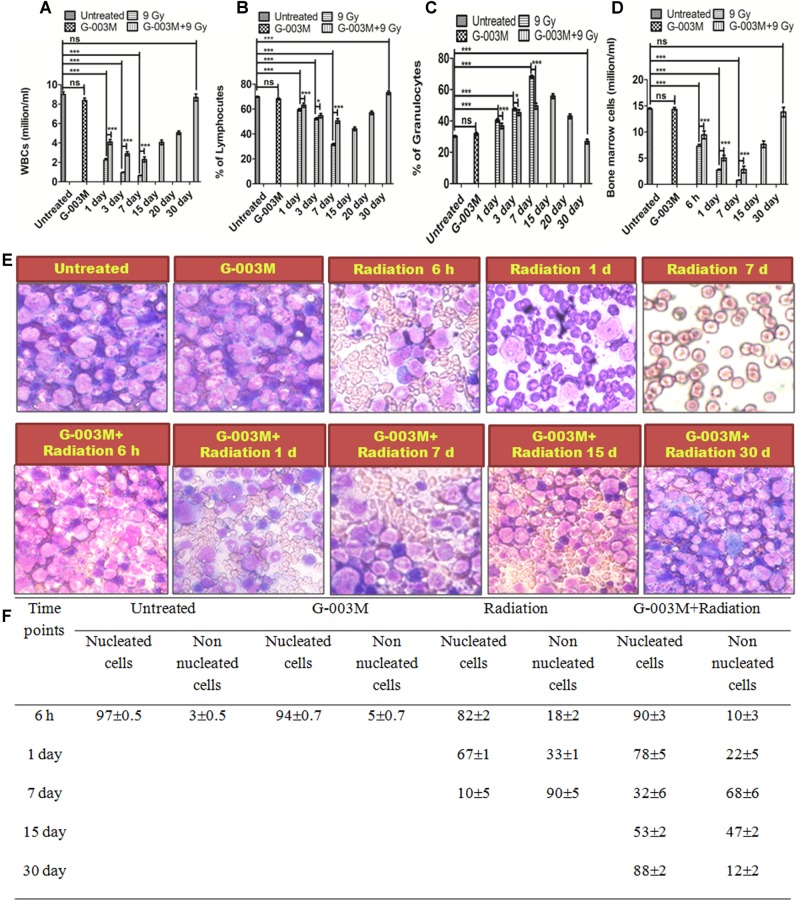
Hematological recovery: **(A)** Graph represents the WBC counts at various time points and treatment groups. **(B)** Graph represents the percentage of lymphocytes at various time points and treatment groups. **(C)** Graph represents the percentage of granulocytes at various time points and treatment groups. **(D)** Graphs represent the bone marrow cell counts at 6 h, 1, 3, 7, 15, and 30 day time intervals in different treatment groups. **(E)** Bone marrow smear of Untreated, G-003M alone, Radiation alone and G-003M+ Radiation were observed under 40X magnification by staining with May-Grünwald Giemsa stain. **(F)** Table shows Effect of G-003M pre-treatment on bone marrow population of lethally irradiated mice at different time intervals. *n* = 6 per group.

Hematopoietic cyto-protective and restorative ability of G-003M was measured by counting the total blood and bone marrow cell numbers at different time intervals for various treatment groups. We have studied the mice blood cell count at 1, 3, 7, 15, and 30 day and the bone marrow counts at 6 h, 1 day, 3 day, 7 day, 15 day, and 30 day time interval. In G-003M alone group the WBC counts and total bone marrow counts were comparable to untreated samples (**Figures 8A–D**). A significant reduction in WBC and bone marrow cell count was recorded up to 7 day post-irradiation compared to untreated controls, however, at later time points the cell counts could not be recorded due to high morbidity or death of animals. In G-003M treated and irradiated groups, significant increase in WBC and bone marrow cells count with respect to radiation at initial time points was noticed while the counts were lower than the untreated group. After the day 7, the cell counts started increasing and at 30^th^ day post-irradiation, the cell counts restored and were comparable to controls. For a better understanding of the G-003M action we also observed the lymphocytes and granulocytes percentage in blood and nucleated and non-nucleated cell (RBCs) in bone marrow cells. We found that lymphocytes and granulocytes percentage was drastically changed in radiation alone and G-003M+irradiated groups as compared to untreated control (**Figures 8E,F**). With the restoration of total blood counts the percentage ratio of lymphocytes and granulocytes also normalized similar to controls.

## Discussion

Development of a radiation countermeasure is highly desirable due to increasing use of nuclear power installations and the heavy use of radioactive isotopes in diagnosis and therapy. Here we evaluated the DNA DSB damage and repair ability of G-003M in the hematopoietic system. Earlier it has been shown to protect the mice hematopoietic (Yashavarddhan et al., 2016; Singh et al., 2017), gastrointestinal (Kalita et al., 2016) and respiratory systems (Saini et al., 2013) against lethal exposures by rendering more than 85% survival (Kalita et al., 2016; Singh et al., 2017). Podophyllotoxin, a component of G-003M, has shown temporary cell-cycle arrest in human lymphoid cells (**Figure 2**) as already shown by many investigators (Sengupta et al., 2005; Dutta and Gupta, 2014) and reversibly binds to tubulin protein resulting in to the arrest of cell-cycle at G2/M phase along with the increased accumulation of Cyclin B1 in the mitosis phase (DiPaola, 2002). We had similar observations in human lymphoid cells treated with podophyllotoxin (**Figure 2**). Docking studies have showed weak interaction of podophyllotoxin with tubulin protein indicative of reversible binding in comparison to colchicines (**Figure 2**) confirming the temporary halt of cell-cycle in G2/M phase permitting DNA to repair. Bioactivity assays further refined the targets involved in biological activities like inflammation, DNA repair, cellular growth, differentiation, neuronal function etc. as shown in Supplementary Table 1, highlighting the relevance in animal’s recovery after lethal irradiation. Rutin, another component of G-003M, scavenged the radiation induced free-radicals in mice blood and bone marrow cells as explained by our results in **Figure 3** and interacted with other biological molecules involved in growth, inflammation, intestinal protection, endocrine function, cell death and DNA repair responsible for radioprotective property of rutin in deciding the fate of any cellular system (Supplementary Table 2).

The hematopoietic protective ability of G-003M was addressed by evaluating the various DNA DSB biomarkers and repair proteins in the blood and bone marrow cells of mice exposed to lethal radiation. DNA DSBs induction rapidly phosphorylates H2AX at serine-139 position to form γH2AX (Rogakou et al., 1998; Sedelnikova et al., 2002) and several reports have shown maximum phosphorylated H2AX foci measured between 30 and 60 min post-irradiation. 53BP1 protein in the chromatin hyper-phosphorylates immediately after sensing the DNA DSBs and relocates at the site of damage along with γH2AX (Tanaka et al., 2006; Luo et al., 2013) and is also reported to facilitate the recruitment of repair factors to damaged DNA sites (Luo et al., 2013). ATM, a PIKK family protein, plays dual role in cell-cycle regulation as well as DNA DSB repair. ATM phosphorylation is maximum between 30-60 min post-irradiation (Osipov et al., 2015). In present investigation, we observed maximum γH2AX, and p53BP1 foci number at 1 h post-irradiation in the mice blood and bone marrow cells and persisting upto 24 h (**Figure 4**). The peak of ATM foci level was similar to γH2AX and p53BP1 at 1 h post-irradiation, however, persisted only upto 6 h post-irradiation (**Figure 5**). The radioprotective formulation G-003M showed significant attenuation of γH2AX, p53BP1 and ATM foci frequency indicating reduced DNA damage in haematopoietic system. This finding was also validated through flow cytometry and Q-PCR study. In corroboration with earlier reports, our results also support the use of γH2AX and p53BP1 as a biomarker of ionizing radiation induced DNA DSBs during radiation accidents (Salassidis et al., 1994; Gupta et al., 2013).

The reduced level of DNA damage markers in both hematopoietic cell types prompted us to evaluate the effect of G-003M on expression level of DNA repair proteins DNA pk, Ku 70/80, Ligase IV, Mre 11, Rad 50 and NBS 1. These repair enzymes are integral part of non-homologous end joining repair pathway. DNA pk is one of the central enzymes of NHEJ involved in DNA DSBs (Weterings and Chen, 2007). It helps in proper alignment of two ends of the damaged DNA and also allows recruitment of various factors for its repair (Jackson, 2002). Ku is an abundant protein complex comprising hetero-dimerized Ku 70 and 80 subunits which are reported to bind at the broken ends of DNA (Britton et al., 2013). It assists in recruitment of nuclease, polymerases and ligases at the site of DNA damage for tethering the DNA ends together (Pannunzio et al., 2014). Ligase IV regulates the fidelity of the DNA DSB repair along with XRCC 4, that acts as a cofactor to stabilize Ligase IV, at the site of broken ends of damaged DNA (Chiruvella et al., 2013). This process plays a critical role in maintaining the genomic integrity, supporting V(D)J recombination and promoting cellular resistance to ionizing radiation. MRN complex, which includes protein Mre 11, Rad 50 and NBS 1 facilitates DNA DSB repair by retaining ATM at the site of DNA DSBs (van den Bosch et al., 2003; Lamarche et al., 2010). MRN complex upon radiation exposure rapidly associates with the DNA DSBs and remains at these sites until the damage is repaired (Nelms et al., 1998). In present investigation the maximum number of DNA pk and ku 80 foci in blood and bone marrow cells was observed at 1 h and persisted only upto the 6 h post-irradiation. However, significant increase in number of DNA repair foci in G-003M treated and irradiated groups than irradiated alone clearly indicates the enhanced DNA repair (**Figure 6**). Our observations with Ligase IV indicated similar pattern to DNA pk and ku 80 with the maximum expression at 1 h post-irradiation. G-003M pre-treatment led to an enhancement in the DNA DSBs repair through an increase in the expression of Ligase IV as compared to the radiation alone group (**Figure 6**). G-003M up-regulates the expression of MRN complex in G-003M pre-treated group when compared with the radiation alone and thus enhances the DNA DSB repair (**Figure 6**). We further used Q-PCR to validate the levels of ATM, Ku 80, Ligase IV and Mre 11 foci as seen through immunostaining. Overall, G-003M accelerates the radiation induced DNA DSBs repair in haematopoietic system by exhibiting increased expression level of NHEJ pathway repair proteins DNA pk, Ku 80, Ligase IV, Mre 11, Rad 50 and NBS 1 resulting in to reduced DNA damage.

Completion of the DNA repair process initiates the apoptosis where the cells with un-repaired or mis-repaired DNA receive programmed cell death signals (Zhang et al., 2014). With this properly functional process most of the damaged cells are eliminated from the body within 24 h post-irradiation (Hunter et al., 2009). Results of our DNA fragmentation study showed maximum fragmentation at 6 h post-irradiation. G-003M pre-treated groups showed significantly reduced fragmentation as shown in **Figure 7** indicating the apoptosis inhibitory effects of the formulation. Along with the nuclear DNA fragmentation several proteins were also involved which regulate cell death. Role of BCL 2 protein levels in determination of survival or apoptosis after radiation or stress exposure is shown earlier (Adams and Cory, 2007). A number of reports have clearly indicated the anti-apoptotic properties of BCL 2 protein which also interact with tumor suppressor P53 and initiate a number of parallel and serial signaling events in response to DNA damage (Fridman and Lowe, 2003). Caspase 3 is another important protein family member widely known for its involvement in cell death and survival (Porter and Jänicke, 1999). G-003M treated and irradiated groups have shown the down-regulation of apoptotic proteins BAX, Caspase 3 and P53 and up-regulation of anti-apoptotic protein BCL 2 at all the time points like 3, 6, and 24 h, showing its role in the radiation induced apoptosis inhibition in the blood and bone marrow cells of mice (**Figure 7**). Effect of apoptosis also manifested in the blood and bone marrow cells with continuous loss in cell number after 24 h post-irradiation and persisted until the animals in radiation alone group died. However, G-003M pre-treated groups showed a significantly reduced cell loss at all time points which could be seen upto 15 days and beyond that the restoration process started leading to full recovery of animals by the 30^th^ day post-irradiation (**Figure 8**). We have also observed the changes in the differential cell counting in blood and bone marrow cells which confirmed radiation induced damage to the bone marrow nucleated cells. Appearance of large number of non-nucleated cells as RBCs at later time points in radiation alone group indicates vascular damage. G-003M pre-treatment restored radiation induced bone marrow toxicity and enhanced the cell count which could be linked to the protection of the stem cells. Similarly, in Differential Leukocyte Counts (DLCs) changes in lymphocytes and neutrophils population was observed in case of radiation alone group that normalized on 30^th^ day in case of G-003M pre-treated group (**Figure 8**).

Our study suggests that G-003M has the potential to reduce the cellular damage by scavenging the free-radicals, arrest the cell-cycle and enhance DNA repair in the hematopoietic system. Based on our encouraging pre-clinical results we will be initiating the clinical trials of this radioprotector.

## Author Contributions

Conceived, designed, and performed the experiments: MY and SS. Analyzed the data: MY, PC, and SS. Contributed reagents/materials/analysis tool: NS and JJ. Wrote the paper: SS, MS, and MG. Manuscript editing: PC.

## Conflict of Interest Statement

The authors declare that the research was conducted in the absence of any commercial or financial relationships that could be construed as a potential conflict of interest.
