# Supplementary material for: Targeting DNA Repair through Podophyllotoxin and Rutin Formulation in Hematopoietic Radioprotection: An in Silico, in Vitro, and in Vivo Study
Source: Front Pharmacol. 2017 Oct 31;8:750. doi: 10.3389/fphar.2017.00750 (PMC5671582; doi:10.3389/fphar.2017.00750)
Supplement: Supplementary file 1 [file Table_1.DOCX]

Supplementary Material

**Targeting DNA Repair through Podophyllotoxin and Rutin Formulation in Hematopoietic Radioprotection: An *In-Silico, In-Vitro and In-Vivo* Study**

M. H. Yashavarddhan^1,2^, Sandeep Kumar Shukla^1^*, Pankaj Chaudhary^3^, Nitya Nand Srivastava^4^, Jayadev Joshi^1^, Mrutyunjay Suar^2^, Manju Lata Gupta^1^

***Correspondence:** Dr. Sandeep Kumar Shukla

**E-mail:** [sandeepshukla@inmas.drdo.in](mailto:sandeepshukla@inmas.drdo.in)

## Supplementary Tables

**Supplementary Table 1. Active targets of podophyllotoxin and role in radiation response.**

| **Targets** | **Role in radiation response** |
| --- | --- |
| Transcription factor p65 isoform 1 [Homo sapiens] | Inflammation, immunity, differentiation, cell growth, tumorigenesis and apoptosis |
| Steroidogenic factor 1 [Homo sapiens] | Endocrine function, cell development, cell differentiation and cell signaling |
| 5-hydroxytryptamine receptor 1E | Neuronal Function |
| Nuclear receptor ROR-alpha | Inflammation |
| STAT3 | Cell growth and apoptosis |
| Cytochrome P450 3A4 isoform 1 [Homo sapiens] | Liver Function |
| Intestinal-type alkaline phosphatase precursor [Bos taurus] | Intestinal Protection |
| Nuclear factor NF-kappa-B p105 subunit isoform 1 [Homo sapiens] | Inflammation, immunity, differentiation, cell growth, tumorigenesis and apoptosis. |
| Amyloid precursor protein | Neuronal Protection |
| Janus kinase 2 (a protein tyrosine kinase) [Homo sapiens] | Cell growth and apoptosis |
| Kruppel-like factor 5 [Homo sapiens] | Intestinal epithelial proliferation, intestinal stem cells, and DNA damage repair |
| Hsf1 protein [Mus musculus] | Cell Cycle check point and repair |
| Serine/threonine kinase 33 [Homo sapiens] | DNA repair, survival and proliferation |
| Protein Mdm4 isoform 1 [Homo sapiens] | DNA damage response cell proliferation and apoptosis |
| E3 ubiquitin-protein ligase Mdm2 isoform a [Homo sapiens] | Mitotic regulator, DNA damage response cell proliferation and apoptosis |
| Microphthalmia-associated transcription factor [Homo sapiens] | DNA damage response, Cell differentiation, proliferation and survival |
| Vma11p [Saccharomyces cerevisiae S288c] | N.A |
| Breast cancer type 1 susceptibility protein | DNA repair and cancer |
| BRCA1-associated RING domain protein 1 | DNA repair and cancer |
| Cystic fibrosis trans-membrane conductance regulator [Homo sapiens] | Chloride trans-membrane transport |
| Toll-like receptor 3 | Inflammation |
| Vif [Human immunodeficiency virus 1] | NA |
| Peripheral myelin protein 22 [Rattus norvegicus] | Neuronal Function |
| Muscarinic acetylcholine receptor M4 [Homo sapiens] | Cell proliferation, signal transduction and synaptic transmission |
| geminin [Homo sapiens] | Inhibition of DNA replication and Cell cycle control |
| ATPase family AAA domain-containing protein 5 | DNA damage response, ATP binding |
| Member 1 (DAX1) | Regulation of hormone production |
| PAX8 [Homo sapiens] | Functionality of thyroid cells, central nervous system development and kidney development |
| TDP1 protein [Homo sapiens] | DNA repair |
| AR protein [Homo sapiens] | Androgenic receptor signaling, cell proliferation |
| Thyroid hormone receptor beta isoform 2 [Rattus norvegicus] | Regulation of Thyroid hormone |
| Estrogen nuclear receptor alpha [Homo sapiens] | Reproductive and Endocrine |
| Aryl hydrocarbon receptor [Homo sapiens] | Aryl hydrocarbon receptor activity, cell cycle and apoptosis |
| Farnesoid X nuclear receptor [Homo sapiens] | Receptor activity for Steroid and thyroid hormones |
| Vitamin D (1) | NA |
| RAR-related orphan receptor gamma [Mus musculus] | Immune system and apoptosis |
| V-jun sarcoma virus 17 oncogene homolog (avian) [Homo sapiens] | NA |
| Retinoic acid nuclear receptor alpha variant 1 [Homo sapiens] | Receptor activity for retinoic, steroid hormones |
| Lethal factor [Bacillus anthracis str. A2012] | NA |
| Tubulin beta chain | Mitotic arrest |
| Tubulin alpha-1A chain | Mitotic arrest |
| Tubulin beta-2B chain | Mitotic arrest |
| Similar to alpha-tubulin isoform 1 | Mitotic arrest |
| Regulatory protein E2 | NA |
| Tubulin beta-3 chain | Mitotic arrest |
| Tubulin beta-4A chain | Mitotic arrest |
| Tubulin beta-2A chain | Mitotic arrest |
| Tubulin alpha-1C chain | Mitotic arrest |
| Tubulin alpha-1B chain | Mitotic arrest |
| Tubulin alpha-4A chain | Mitotic arrest |
| Tubulin alpha-3C/D chain | Mitotic arrest |
| Tubulin beta-8 chain | Mitotic arrest |
| Tubulin beta-4B chain | Mitotic arrest |
| Tubulin beta-1 chain | Mitotic arrest |
| Tubulin beta-6 chain | mitotic arrest |
| Tubulin alpha-3E chain | Mitotic arrest |
| Cytochrome P450 2C9 | Liver Function |
| Cytochrome P450 3A4 | Liver Function |
| Glucocorticoid receptor | Development, metabolism, and immune response. |
| Cytochrome P450 2C19 | Liver Function |
| Nuclear receptor subfamily 1 | Inflammation, Metabolic function and DNA methylation |
