# Supplementary material for: Targeting DNA Repair through Podophyllotoxin and Rutin Formulation in Hematopoietic Radioprotection: An in Silico, in Vitro, and in Vivo Study
Source: Front Pharmacol. 2017 Oct 31;8:750. doi: 10.3389/fphar.2017.00750 (PMC5671582; doi:10.3389/fphar.2017.00750)
Supplement: Supplementary file 2 [file Table_2.DOCX]

Supplementary Material

**Targeting DNA Repair through Podophyllotoxin and Rutin Formulation in Hematopoietic Radioprotection: An *In-Silico, In-Vitro and In-Vivo* Study**

M. H. Yashavarddhan^1,2^, Sandeep Kumar Shukla^1^*, Pankaj Chaudhary^3^, Nitya Nand Srivastava^4^, Jayadev Joshi^1^, Mrutyunjay Suar^2^, Manju Lata Gupta^1^

***Correspondence:** Dr. Sandeep Kumar Shukla

**E-mail:** [sandeepshukla@inmas.drdo.in](mailto:sandeepshukla@inmas.drdo.in)

## Supplementary Tables

**Supplementary Table 2. Active targets of rutin and role in radiation response.**

| **Targets** | **Role in radiation response** |
| --- | --- |
| Acetylcholinesterase | Neuronal function |
| Aldose reductase | Glucose metabolism |
| Alkaline phosphatase | Maintenance of alakaline phophatase activity |
| Bifunctional epoxide hydrolase 2 | Anti-Inflammatory |
| Crystal Structure Of The Human 2-Oxoglutarate Oxygenase Loc390245 | Histones methylation |
| Dihydrolipoamide dehydrogenase [Homo sapiens] | Mitochondria energy metabolism |
| DNA polymerase beta [Homo sapiens] | DNA repair |
| DNA polymerase kappa [Homo sapiens] | DNA repair |
| Estrogen nuclear receptor alpha [Homo sapiens] | Reproductive and endocrine |
| Eukaryotic translation initiation factor 4H [Homo sapiens] | Regulation of protein synthesis |
| FAD-linked sulfhydryl oxidase ALR [Homo sapiens] | Hepatic protection |
| Glycogen synthase kinase-3 beta isoform 1 [Homo sapiens] | Energy metabolism and neuronal cell development |
| Intestinal alkaline phosphatase precursor [Mus musculus] | Intestinal protection |
| Lysosomal alpha-glucosidase preproprotein [Homo sapiens] | Glucose synthesis from glycogen |
| M18 aspartyl aminopeptidase [Plasmodium falciparum 3D7] | Not identified |
| MPI protein [Homo sapiens] | Glycosylation |
| Neuromedin-U receptor 2 | Regulation of energy balance |
| Nuclear factor NF-kappa-B p100 subunit | Inflammation, immunity, differentiation, cell growth, tumorigenesis and apoptosis. |
| Nuclear factor NF-kappa-B p105 subunit | Inflammation, immunity, differentiation, cell growth, tumorigenesis and apoptosis. |
| Peroxisome proliferator-activated receptor gamma | Neuronal function |
| Polyadenylate-binding protein 1 [Homo sapiens] | Regulation of m-RNA metabolism |
| Serum albumin | Inflammation |
| Sialidase | Anti-microbial |
| Transcription factor p65 | Inflammation, immunity, differentiation, cell growth, tumorigenesis and apoptosis. |
