# Supplementary figures and images for: Targeting DNA Repair through Podophyllotoxin and Rutin Formulation in Hematopoietic Radioprotection: An in Silico, in Vitro, and in Vivo Study
Source: Front Pharmacol. 2017 Oct 31;8:750. doi: 10.3389/fphar.2017.00750 (PMC5671582; doi:10.3389/fphar.2017.00750)

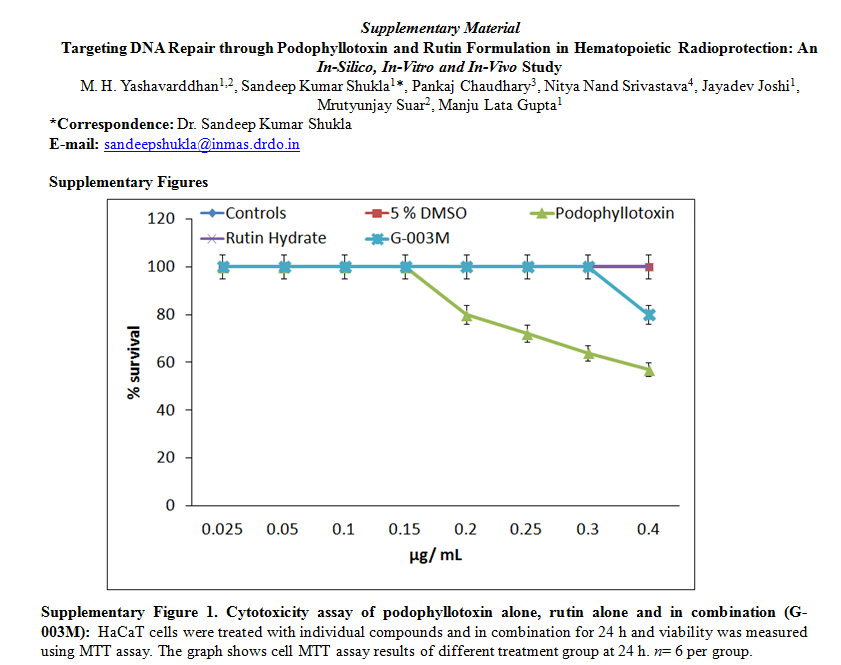

Supplement: Supplementary file 3 [file Image_1.TIF]
